# [Expression of Concern] Src homology phosphotyrosyl phosphatase 2 mediates cisplatin-related drug resistance by inhibiting apoptosis and activating the Ras/PI3K/Akt1/survivin pathway in lung cancer cells

**DOI:** 10.3892/or.2025.8979

**Published:** 2025-08-26

**Authors:** Chunlan Tang, Hu Luo, Dan Luo, Heping Yang, Xiangdong Zhou

Oncol Rep 39: 611–618, 2018; DOI: 10.3892/or.2017.6109

Following the publication of this paper, it was drawn to the Editor's attention by a concerned reader that the immunohistochemical images shown in Figs. 1E and 5E appeared to show an overlapping section, even though Figs. 1 and 5 were intended to show the results of SHP2 and Ras expression experiments, respectively.

The authors were contacted by the Editorial Office to offer an explanation for this apparent anomaly in the presentation of the data in this paper; however, up to this time, no response from them has been forthcoming. Owing to the fact that the Editorial Office has been made aware of potential issues surrounding the scientific integrity of this paper, we are issuing an Expression of Concern to notify readers of this potential problem while the Editorial Office continues to investigate this matter further.

